# Knowing me, knowing you—A study on top-down requirements for compensatory scanning in drivers with homonymous visual field loss

**DOI:** 10.1371/journal.pone.0299129

**Published:** 2024-03-01

**Authors:** Bianca Biebl, Max Kuhn, Franziska Stolle, Jing Xu, Klaus Bengler, Alex R. Bowers

**Affiliations:** 1 Chair of Ergonomics, TUM School of Engineering and Design, Technical University of Munich, Garching, Germany; 2 Schepens Eye Research Institute of Mass Eye and Ear, Department of Ophthalmology, Harvard Medical School, Boston, MA, United States of America; University of Hertfordshire, UNITED KINGDOM

## Abstract

**Objective:**

It is currently still unknown why some drivers with visual field loss can compensate well for their visual impairment while others adopt ineffective strategies. This paper contributes to the methodological investigation of the associated top-down mechanisms and aims at validating a theoretical model on the requirements for successful compensation among drivers with homonymous visual field loss.

**Methods:**

A driving simulator study was conducted with eight participants with homonymous visual field loss and eight participants with normal vision. Participants drove through an urban surrounding and experienced a baseline scenario and scenarios with visual precursors indicating increased likelihoods of crossing hazards. Novel measures for the assessment of the mental model of their visual abilities, the mental model of the driving scene and the perceived attention demand were developed and used to investigate the top-down mechanisms behind attention allocation and hazard avoidance.

**Results:**

Participants with an overestimation of their visual field size tended to prioritize their seeing side over their blind side both in subjective and objective measures. The mental model of the driving scene showed close relations to the subjective and actual attention allocation. While participants with homonymous visual field loss were less anticipatory in their usage of the visual precursors and showed poorer performances compared to participants with normal vision, the results indicate a stronger reliance on top-down mechanism for drivers with visual impairments. A subjective focus on the seeing side or on near peripheries more frequently led to bad performances in terms of collisions with crossing cyclists.

**Conclusion:**

The study yielded promising indicators for the potential of novel measures to elucidate top-down mechanisms in drivers with homonymous visual field loss. Furthermore, the results largely support the model of requirements for successful compensatory scanning. The findings highlight the importance of individualized interventions and driver assistance systems tailored to address these mechanisms.

## Introduction

### Driving with homonymous visual field loss

Driving requires the complex integration of cognitive, visual, and motor abilities. One of the most important modalities is vision since 90% of the information relevant to driving is perceived visually [1]. Therefore, a significant reduction of vision leads to a loss of the driving license and reduction of individual mobility in many countries [2, 3]. Homonymous visual field loss (HVFL) describes a loss of vision in the same half of the visual field in both eyes, resulting from lesions to the postchiasmal visual pathways. The most common causes are stroke and traumatic brain injury [4]. Homonymous hemianopia, the loss of one complete hemifield, is the most severe type of HVFL. Hemianopia causes difficulties in various aspects of driving. Both on-road and driving simulator studies have found issues regarding lateral vehicle guidance with a deviated lane position and increased steering instability [5–7], longitudinal vehicle guidance including difficulties with appropriate speed adaptation and gap estimation [5, 8, 9], and hazard avoidance [10–12]. One of the main challenges contributing to all other problem areas is inadequate scanning. Prior studies have reported that some drivers with hemianopia scan well and some show insufficient scanning behavior, characterized by generally smaller gaze and head movements as well as slower, later, and less precise movements, especially in and toward the blind visual field [5, 6, 10, 13–15]. These scanning issues can be explained by considering the SEEV model [16]. The SEEV model describes the relationship of factors contributing to attention allocation. These factors are divided into top-down and bottom-up processes. The bottom-up factors saliency and effort describe stimuli-based processes that draw attention toward objects or areas of interest (AOI). Although a higher saliency of the AOI’s sensory characteristics encourages a gaze shift toward this AOI, a greater mental or physical effort reduces the likelihood of an attention shift. On the contrary, top-down factors are influenced by higher-order cognitive processes in connection with content from long-term memory. In the SEEV model, those are the expectancy, i.e., the likelihood of new information in the AOI and the value of this information for successful completion of the task at hand and the value of the task itself.

### Compensation of visual field loss

Biebl et al. [17] summarized how the processes of attention allocation are influenced by homonymous visual field loss. They stated that compensation requires a stronger reliance on top-down processes to compensate for the missing bottom-up input and requirement for larger gaze shifts towards the periphery. Compensation describes all measures taken to reduce negative influences of an impairment on a task of daily living [18]. Baltes and Graf [19] proposed a threefold differentiation between selection, optimization, and compensation. Compensation covers the same type of behavior that Michon [20] calls tactical compensation, which describes actions taken within a particular situation to counteract the impact of the impairment at that moment. Selection parallels the term strategic compensation proposed by Michon [20]. It refers to all actions taken before entering a situation, e.g., choosing a less busy or complex type of road or avoiding driving during rush hour, rain, or darkness. While both types of compensation are valuable, we focus on tactical compensation as it is most safety-critical when considering hazard avoidance in everyday driving since hazards can arrive at any moment. Previous research suggests that some drivers with HVFL can compensate for their impairments so that their driving performance resembles those of normal-sighted drivers. The proportion of drivers able to compensate well varies between 14% and 77% due to differing sample characteristics and methodologies [5, 8, 21, 22]. However, it remains unclear why some people can compensate for large visual field impairments, and others cannot. There have been several reports on potential influencing factors. However, most investigations do not apply a systematic but rather a coincidental approach. Discussed personal factors are age [18], cognitive status [18], expectations [23], prior experiences [13] and time since onset [18]. The systematic effect of the impairment’s location, side, and extent is widely disputed [5, 10]. The situational factors investigated encompass the complexity or task demand [24, 25], movement of collision objects [12, 26], and the existence of lane markings [22]. Properly classifying or predicting the compensatory strategies and driving ability of persons with HVFL may guide the development of trainings and assistance systems and inform the often restrictive legislations adjudicating the allocation of driving licenses to persons with visual impairments. Understanding the mechanisms behind successful compensation can support these processes to achieve inclusive mobility.

### Mental models

To decipher the mechanisms underlying tactical compensation, Biebl et al. [17] analyzed which aspects of attention allocation are altered for drivers with HVFL and inferred requirements to compensate for these challenges. They conclude that the disturbance of bottom-up mechanisms for attention allocation means that drivers with HVFL must make larger gaze shifts to the blind side without peripheral information to guide scanning movements or to draw attention to salient hazards. They emphasize the importance of top-down processes and, most importantly, the existence and usage of correct and extensive mental models to account for those challenges. The term mental model refers to a person’s internal representation of the external world that is continuously formed and developed by perceiving and interacting with the world [28]. Stored in long-term memory, mental models are frameworks that help humans describe and understand their sensory input, put it into reference, anticipate future states, and guide action planning [27]. They can, therefore, also aid attention allocation, as the top-down mechanisms expectancy and value from the SEEV model represent aspects of the mental model of AOIs in the current scene. It should be noted that mental models do not have to be correct and mostly only represent a diffuse picture and a limited section of the actual relations, depending on individual experiences, motives, and goals [27–30]. Mental models have extensively been investigated in human-computer interaction research to describe a human’s understanding or representation of a technical system [30]. However, mental models have also been studied concerning more abstract topics like the perceived time benefit of speed changes [28]. Although mental models have received growing attention in all human factors research, there are no gold standards for their measurement. Gaspar et al. [31] summarized three approaches to measuring mental models: observational methods, verbal or written reports, and survey questions. In further literature, a variety of methods can be found, among others, thinking aloud, interviews, written reports, questionnaires, content analysis, concept mapping, card sorting, or pairwise ratings [27, 32–35]. While all of these metrics can be more suitable for one or the other research question, many come with limitations that warrant careful interpretation of the results.

### Theory on the requirements for compensatory scanning

**Fig 1:**
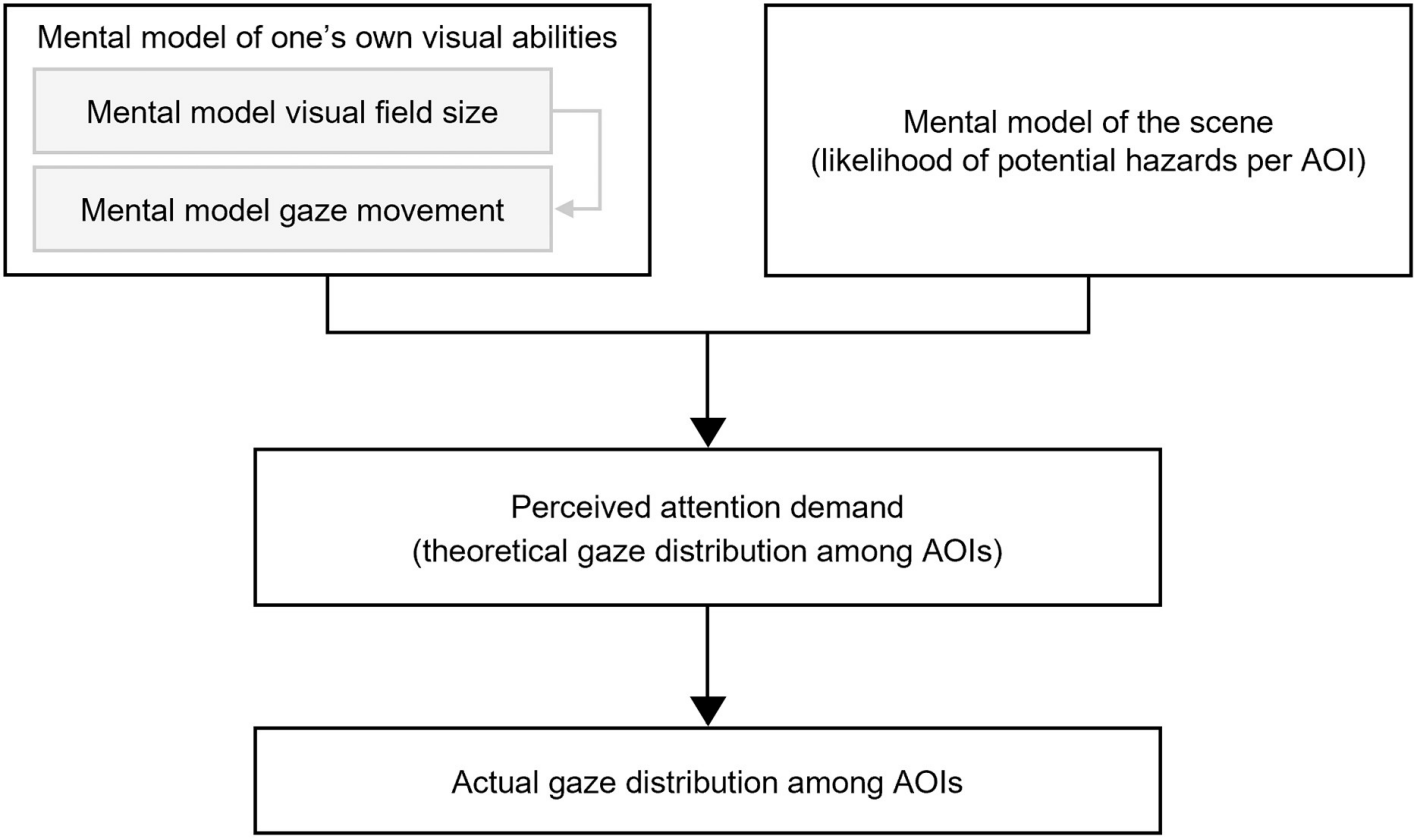
Theoretical model for the requirements for compensatory scanning.

The mental models relevant to compensation proposed by Biebl et al. [17] are the mental model of one’s visual abilities and the mental model of the driving scene. First, the authors argue that drivers need to be aware of their visual field loss and its exact measurements as a spatial internal representation of their visual abilities to calculate the gaze movements required to perceive an AOI where relevant information is assumed. Therefore, an appropriate mental model of visual abilities can counteract the lack of peripheral input to guide gaze shifts to the blind side and support adaptation to the demand for larger gaze scans to the blind side. As shown in Fig 1, the mental model of vision can be differentiated into the internal representation of the size of the visual field loss and the gaze movement required to perceive objects in space. It should be noted that the original definition of mental models refers to presentations of the outside world and must be understood in a broader sense here to also include visuospatial processes within oneself. Secondly, drivers with HVFL require an appropriate mental model of the driving scene according to Biebl et al. [17], which can be regarded as an aspect of situation awareness. Situation awareness or the situation model according to Endsley [36] has three levels representing different stages of forming an overall situation awareness. Level 1 refers to the perception of the scene and represents the basis for all further levels. Level 2, comprehension, entails the processing of this information in terms of recognition, designation and putting into context. Lastly, projection in level 3 entails the anticipation of future states of the object or scene as the basis for action planning and execution. In this situation awareness model, mental models direct attention toward objects or AOIs at level 1 and influence level 2 and 3 by applying knowledge, prior data, as well as schemata for information processing [37]. The mental model of the driving scene as described by Biebl et al. [17] refers to such mental models with a focus on the internal representation of the likelihood of hazards within the periphery. The combination of both the mental model of one’s visual abilities and of the scene is assumed to be the basis for the perceived attention demand in the different AOIs which in turn is the foundation for the actual attention ratio when driving (see Fig 1).

### Scope

The theoretical model depicted in Fig 1 is based on theoretical considerations using models of information processing and attention as well as reports from prior on-road and driving simulator studies. The investigation of the processes underlying compensatory scanning and the verification of this model has the potential to elucidate the great individuality of compensatory abilities and strategies [38]. This would shed light on the long-asked questions why some drivers with HVFL can compensate well while others cannot, and which factors influence this differentiation. This paper aims at contributing to this objective. The first goal was the development of appropriate methods to measure the mental model of the visual abilities, the mental model of the driving scene and the perceived attention demand (see Fig 1). These methods were then integrated in a study protocol to test their feasibility. The second aim of the pilot study presented in this paper was to use the data from these new measures to get insights into the mental models and the top-down driven scanning strategies of drivers with HVFL compared to those with normal vision (NV). Lastly, the connection between these top-down mechanisms and their relation to the actual scanning behavior and driving safety were used to screen the model’s suitability and guide future studies for the model’s further verification. All three aims were achieved and produced valuable findings for future research.

## Materials and methods

### Sample

The study was conducted at Schepens Eye Research Institute in Boston, MA, between July 22, 2022, and November 1, 2022. In total, 25 participants took part in the study, 16 of whom were included in the final data set. Participants were excluded due to a lack of prior driving experience, indications of visual neglect (Bells test), or cognitive impairments (<13 in Montreal Cognitive Assessment short form). The final sample consisted of eight participants with homonymous visual field loss (HVFL) and eight age- and gender-matched participants with normal vision (NV) to provide comparison data. At the time of the study, the mean age of the participants was 50.81 years (SD = 16.74 years, Min = 25 years, Max = 83 years) and two were female. One participant had left-sided (HVFL008), and one participant had right-sided (HVFL013) homonymous quadrantanopia. The other six participants in the HVFL group were equally distributed among left-sided (HVFL003, 007, 011) and right-sided (HVFL001, 002, 006) homonymous hemianopia. Two out of eight participants with HVFL stated that they drove regularly (HVFL007 and 008), while all NV participants drove regularly. Three participants with HVFL took part in road traffic as cyclists (HVFL 003, 008, 011), one of which (HVFL003) cycled daily. All participants had a visual acuity of at least 20/40 in each eye and no motor impairments that affected driving.

### Driving simulator

**Fig 2:**
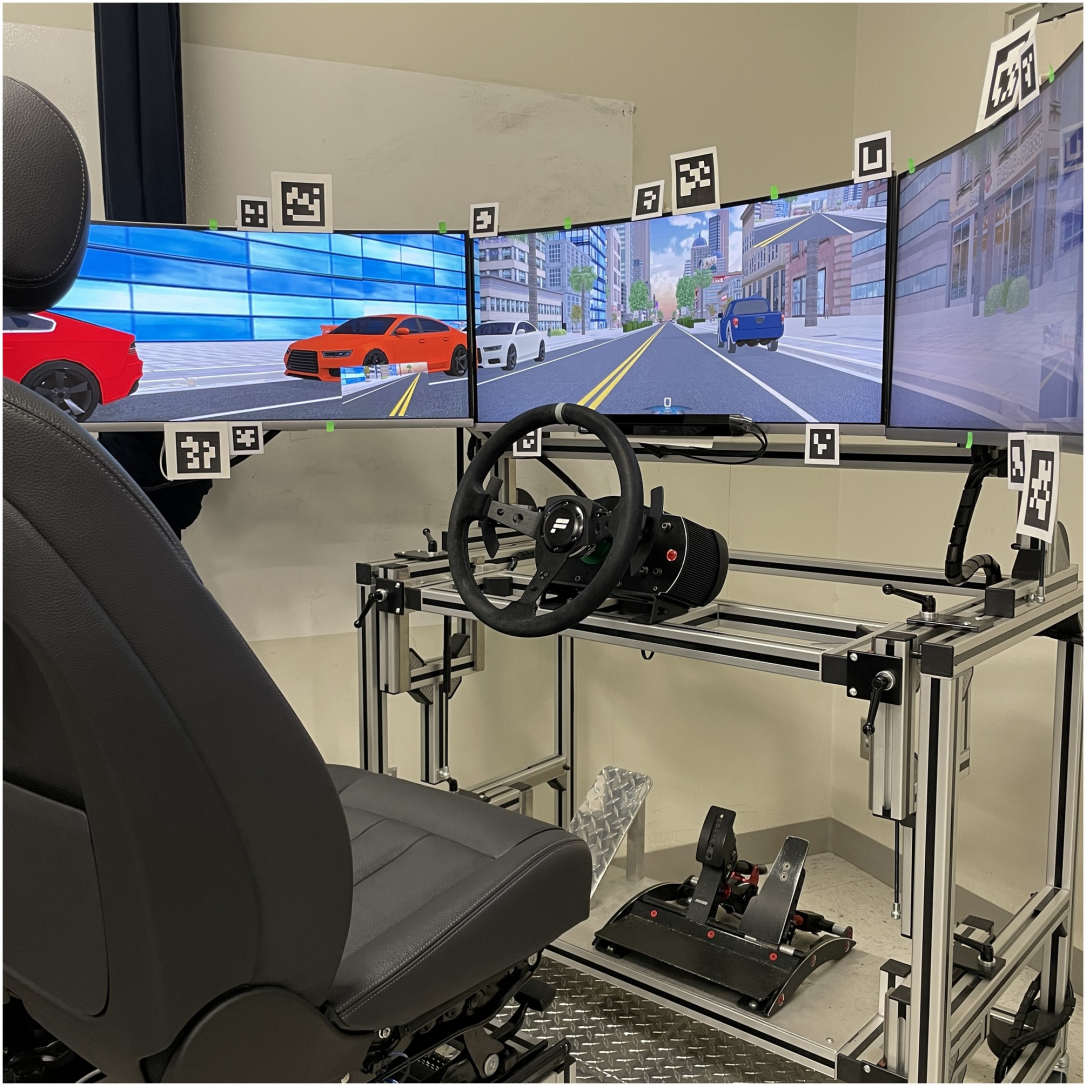
Driving simulator.

A fixed-base, custom-built driving simulator (see Fig 2) was used which included three curved monitors (Samsung CF791, curvature 1500R, 34”, ratio 21:9, 3440 x 1440 pixels resolution) making up a horizontal field-of-view of 180° with 4 ms response time. The participants were seated in an adjustable car seat. They controlled the simulated ego vehicle with a steering wheel (Fanatec ClubSport Wheel Base V2, 900° rotation) and standard gas and brake pedals (Fanatec ClubSport Pedals V3, automatic transmission). The driving simulation was implemented and run with Unity (2018.3.34f1 (64-bit); [39]). Eye tracking was performed with the head-mounted Dikablis Glasses 3, including a frontal scene camera (1920 x 1080 pixel resolution at 30 Hz) and two eye cameras using infrared sensors (648 x 488 pixels). The markers for the AOI definition in the frontal scene were positioned evenly surrounding the three screens.

### Experimental track

**Fig 3:**
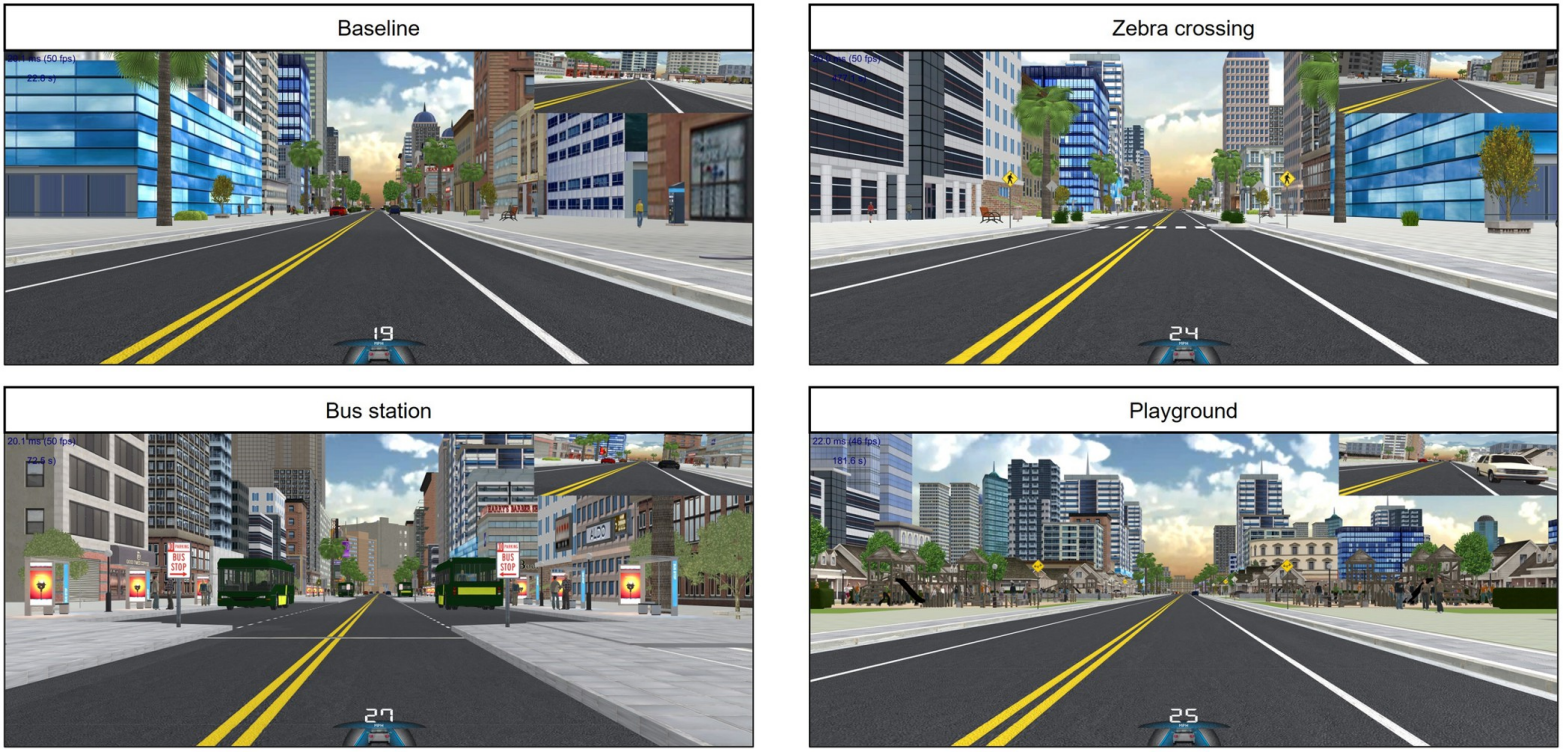
Scenarios.

The participants performed four drives in total, one acclimatization drive to get used to the control of the driving simulator and three experimental drives. All experimental drives were set in an urban surrounding with broad sidewalks, little traffic, and a speed limit of 25 mph. Each drive took approximately 8 to 10 minutes and only differed in the order of the scenarios. Participants drove through intersections and curves that will not be discussed further here and multiple straight road segments. In experimental drives, participants encountered four scenarios on straight-road segments (see Fig 3): a scenario without any situational markers (used as a baseline) and three scenarios with situational markers (a zebra-crossing scenario, a bus-station scenario, and a playground scenario). These situational markers were chosen to be so-called foreshadowing events [40, 41] or situational precursors [42], that can serve drivers with highly developed mental models to better anticipate the hazards they point toward. In the present study, they were used to introduce variance in the mental models of the scene to evaluate the proposed connections in the theoretical model. In addition, they allow an interpretation of drivers’ adaptability to situations with different hazard likelihoods. While the zebra crossing was depicted purely through the road infrastructure, namely lane markings and road signs, the bus station additionally introduced another traffic participant as a precursor stimulus, and the playground hinted at an increased risk of hazards by vulnerable road users moving in the periphery. All precursor scenarios were symmetrical and had centrally displayed information via road signs to be suitable for drivers with either right- or left-sided field loss and give no indication of the side of a potential hazard. The participants experienced each scenario (baseline, zebra crossing, bus station, playground) without a hazard, with a hazard crossing the road from the left side and a hazard crossing the road from the right side to evaluate hazard avoidance. Each condition of the precursor scenarios occurred once, and the scenarios were evenly distributed among the three drives. All conditions of the baseline (no hazard, hazard left, hazard right) occurred three times (once per drive), so that each precursor scenario could be compared with the same condition as the baseline in the same drive to account for potential learning or order effects. The drives were permuted. The hazard was a cyclist that appeared with a time to collision of 5 seconds at an eccentricity of 30°. When the participants held their speed constant after the onset of the cyclist, a collision occurred. The cyclist disappeared in case of a collision to avoid emotional disturbance of the participants or learning effects when disruptive collisions are experienced.

### Materials and measures

Standardized measures were used to assess the binocular visual field size using a Goldman Perimeter 940 (V4e target), monocular visual acuity using the Freiburg Vision Test FrACT [43], visual neglect using Bells test [44], and cognitive decline using the Montreal Cognitive Assessment MoCA [45]. The measured visual field size was used as comparison value for each participant’s mental model of their field extent. The other measures were used to check the participant’s eligibility to take part in the study. After each drive in the driving simulator, participants were asked to rate their subjective well-being and confidence in their control of the car on a scale from 0 to 20 (best rating) to ensure that participants were fit for the upcoming drive. Extensive questionnaires were designed to retrieve additional information on the participants’ demographic background, medical history, driving experience, and subjective perception of their HVFL, tactical and strategic compensation, and driving safety.

### Existent approaches for measuring mental models

New methods were required to measure the mental model of vision, the mental model of the driving scene and the subjective perceived attention demand. Measuring mental models is a recurring issue in many domains [27]. Rouse and Morris [27] distinguish between empirical experiments, empirical modeling, analytical modeling, and verbal reports. Empirical experiments parallel one of the three types of measures for mental models proposed by Gaspar et al. [31]. This approach follows the consensus that mental models can be inferred by observing indicative behavioral markers and has a long history in research on monitoring performance [29, 46–49]. However, it can be argued that these approaches are suitable for simpler and more controlled laboratory-based settings. Additional processes like, e.g., workload and attention management in more complex situations like driving can disrupt the translation from mental model to behavior. Another approach Rouse and Morris [27] and Gaspar et al. [31] mention is verbalizing the mental model either during or after the task. The benefit of all measurements taken during the task is the proximity to the relevant stimuli that activate the mental models [50]. However, thinking aloud is unsuitable for our setup since talking is rather slow and time-consuming compared to the high speed of scanning movements and the quick development and succession of relevant driving scenes. Verbalization during the drive might distract from driving, be delayed, or point toward certain aspects that would otherwise go unnoticed [51]. Freezing the screen before asking questions eliminates this issue, as done in Endsley’s SAGAT method for measuring situation awareness [36]. However, it should be noted that this disruption of the drive disables usage of the actual gaze data. Regarding the type of verbal report, open questions allow for an unbiased exploration. However, they will only elicit those aspects of the mental model that the participant pulls from the long-term memory into working memory or thinks the instructor is asking for [27]. Additionally, discrepancies between verbalized thought and performed action may be observed [27, 52].

### Measurement of mental model of the driving scene and the perceived attention demand

**Fig 4:**
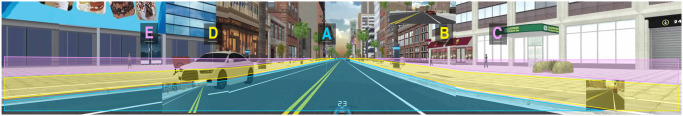
Images used for the measurement of the mental model of the driving scene and the perceived attention demand. The five AOIs were marked as overlay in different colors: left far periphery (E, pink), near left periphery (D, yellow), center (A, blue), near right periphery (B, yellow), and far right periphery (C, pink). The same AOIs were marked in each situation: baseline (depicted here), zebra crossing, bus station, and playground. The small, inserted images on the left and right provided the views from the side-mirrors.

To diminish influences on the actual driving and scanning behavior, we opted for a version of freezing the scene as proposed by Endsley [51], where after completion of the driving simulator part of the study, participants were presented with videos of the different driving scenes they had just experienced in the experimental drives. The driving simulation video was viewed in full-screen mode on all three screens of the driving simulator to emulate a similar setup and elicit a mental model close to the one active during the drive. Each video was about 8 seconds, which should suffice to create an inner representation of a current driving scene [53, 54]. Videos from all scenes (baseline, zebra crossing, bus station, playground) were shown without a crossing hazard. The video stopped 3 seconds before reaching the collision zone, so the participants were still in the final approach phase. The video ended on a black screen. Since the processing time of stimuli plays a significant role in forming situation awareness and activating mental models, reducing the time of stimuli being present can help highlight differences in inner representations [55]. According to the authors, prolonged exposure to the image could help those with weaker mental models fill the gaps with additional information processing in the time that was not available during the drive. After presenting the video, participants were asked whether they remembered experiencing this scenario during the drive to check level 1 of situation awareness. The second question asked participants to describe the scenario to evaluate level 2 of situation awareness. When participants only mentioned irrelevant aspects of the driving scene (e.g., “straight road” without any situational markers), the instructor asked for more detail. The third question targeted the mental model of the driving scene, representing an aspect of level 3 situation awareness. For this question, the last image of the video was shown with five marked AOIs (as depicted in Fig 4). The participants were asked to indicate on a 6-point Likert scale (“very likely” to “very unlikely”) the likelihood of information about potential collision objects being found in each of the respective areas. Participants with HVFL were reminded that the likelihood of the hazard should be evaluated without regards to their visual field loss. According to the theory in Fig 1, the perceived attention demand results from one’s representation of the likelihood of hazards within the scene combined with the awareness of one’s visual abilities and is the top-down basis for the actual attention allocation. The question for this construct was based on the same setup as the measurement of the mental model of the driving scene but with a different task. To emphasize the greater abstraction level of this scene, the still image with the marked AOIs was minified to only be presented on the center screen. Participants were asked to rank the AOIs with respect to the amount of attention necessary in each AOI to complete the driving task. All questions were asked before showing the video of the next scenario.

### Measurement of the mental model of vision

**Fig 5:**
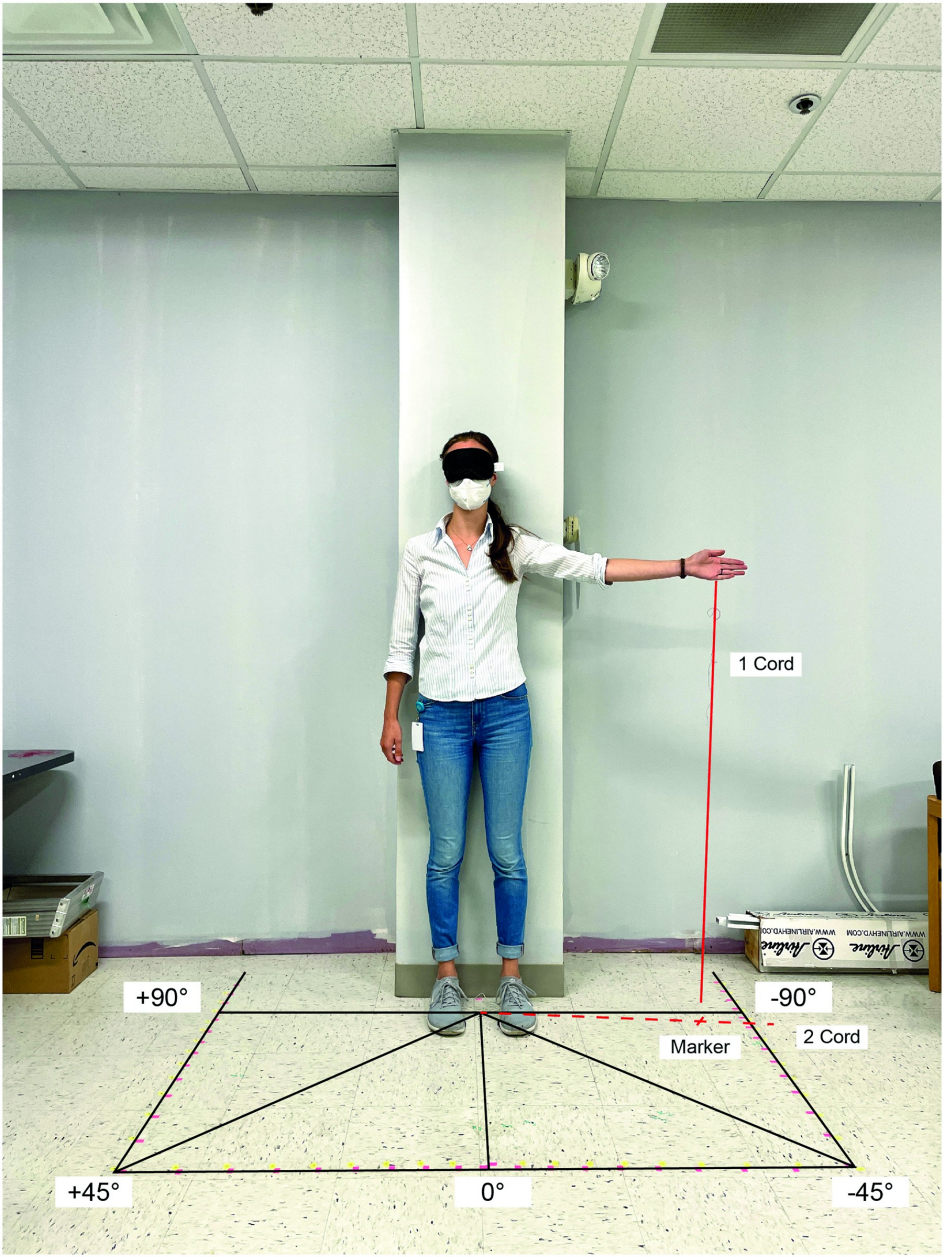
Setup for the measurement of the mental model of vision.

The mental model of vision is a spatial representation, so verbalizations might lead to distortions or misrepresentations [27, 56]. On the other hand, visualizations are also inappropriate, as participants with HVFL only perceive a limited section of any image presented to them. Therefore, we designed a test solely based on participants’ space perception (see Fig 5). Participants stood in front of a wall in a fixed position with blindfolded eyes to eliminate any visual input. Then, they had to indicate different horizontal eccentricities in their visual field by pointing toward this direction with the ipsilateral outstretched arm and index finger. A long string attached to this index finger allowed the instructor to indicate the position on the floor, which had marks for the respective eccentricity with a range of 110° toward the left and right sides. Participants had to indicate the following positions:

- The center midline with the left and right hands, respectively.
- On the blind side: The border between the intact visual field and the blind field.
- On the seeing side: The peripheral end of the visual field.

The participants were instructed to start each trial with their arms stretched straight ahead and to keep the trunk and head straight. For the mental model of the required gaze movements, an object was positioned in the blind field at an eccentricity of 30°. After this object was pointed out and the HVFL participants looked at it, they had to point at the gaze position required to perceive this object with blindfolded eyes.

### Gaze distribution and driving performance

The last construct in the theoretical model in Fig 1 is the gaze distribution as the behavioral outcome of the top-down processes. It was represented by the attention ratio (percentage of time the gaze lies within an AOI during a defined time frame). The attention ratio was calculated for each of the five AOIs in Fig 3 that were also used to evaluate the mental model of the driving scene and the perceived attention demand, so that potential relations between these constructs were directly represented. The considered time frame entailed the time between the cyclist’s onset and the collision zone’s entry. At target speed, this took 5 seconds with the still image used for the subjective measurements lying in the middle of that time frame. We only included scenarios without the hazard since detecting the cyclist would alter the subsequent gaze behavior. The scenarios with hazards from either side were used to identify collisions or critical interactions with the cyclist to indicate the driving performance. While the performance is not directly part of the model in Fig 1, it can be argued that a successful application of top-down strategies and good compensatory scanning should lead to high performances as final outcome of the model. Situations were rated as critical if the minimal post-encroachment time was below 1 second [57, 58].

### Procedure and study design

The study protocol followed the Declaration of Helsinki and was approved by the institutional review board at Mass General Brigham (IRB protocol 2019P001714). After arriving at the Schepens Eye Research Institute laboratories, the participants received information about the study and gave their written informed consent to participate. After a demographic questionnaire and the acclimatization drive, the Dikablis glasses were calibrated. If participants removed the glasses between the three following experimental drives, calibration was repeated. Participants had to indicate their physical comfort and subjective ability to control the car after each drive. The experiment was paused as necessary if the score on either question fell below 10. Directly after the third drive, the mental model of the driving scene, the perceived attention demand, and the mental model of vision were measured. The session was concluded with additional questionnaires and tests on cognitive and visual abilities. Participants were reimbursed for time and travel expenses. The visual abilities of the participants served as the between-subject factor (HVFL; NV). The scenarios (baseline, zebra crossing, bus station, playground) served as the within-subject factor in the measurement of the gaze movements and performance during the drive, the mental model of the scene and the perceived attention demand. The mental model of the visual field size was measured in all subjects, the mental model of the required gaze movements was only measured for HVFL participants as this method was not applicable for participants with full vision.

### Data processing and analysis

#### Mental model of vision

All indicated eccentricities regarding the mental model of vision were centered on the position of the subjective central midline indicated by the same arm since this value fluctuated around 0° between participants and between the left and right arms. The mental model of the size of the visual field was evaluated by calculating the difference between the subjective and the objective extent of the visual field on both sides. The same was done for the mental model of the required gaze movements. The latter had one missing data point.

#### Mental model of the driving scene

The hazard likelihoods assigned to each AOI in the measurement of the mental model of the driving scene were graphically evaluated per scenario by plotting and connecting the AOI evaluations from left to right. We additionally calculated the median likelihood over all AOIs per scenario and participant. To check the connection between the mental model of the driving scene and other measures, we additionally allocated and plotted the rank order of the AOIs according to their hazard likelihood, whereby the same ranks were possible for multiple AOIs.

#### Perceived attention demand

The measurement of the perceived attention demand produced a ranked order of all five AOIs per scenario, whereby no two AOIs received the same rank. This order was viewed graphically by plotting and connecting the AOIs from highest to lowest rank.

#### Attention ratio

A similar rank plot was drawn for the attention ratio, whereby the same ranks were possible for multiple AOIs. The attention ratio was measured as a continuous variable and provided more detail than the subjective measures. To not overinterpret minor differences between the attention ratio between AOIs (e.g., 10.00% and 10.10% in two AOIs), any differences smaller than 1% were discarded. To verify the connection of the mental model of vision with other constructs in the theoretical model we evaluated whether the blind side was prioritized over the seeing side (received higher ratings, higher ranks, or a higher attention ratio) by the HVFL participants.

#### Connections between constructs

The connection between all AOI-based measures (the mental model of the driving scene, the perceived attention demand, and the attention ratio) was made by checking the similarity of the AOI rankings. In each measure, five AOIs could be ranked according to their allocated hazard likelihood, attention demand rank, or attention ratio. Those five AOIs could be broken down into ten pairs of two AOIs each. We compared each pair between two measures of interest and checked if the order was identical (e.g., whether the left far periphery was ranked higher than the near left periphery in the perceived attention demand and the actual attention allocation). This resulted in a maximum match score of 10. A mean match was calculated for all participants per scenario and connection (mental model of the driving scene vs. perceived attention demand; perceived attention demand vs. attention ratio). Three data points per participant were available for the baseline scenario for the attention ratio since it was experienced in all three drives, and the mean was calculated over all available data points. There was one missing data point in the mental mode of the scene for the far left AOI. The match with the perceived attention demand was extrapolated from the available six matches between the remaining four AOIs to the maximum of 10 matches in all other cases. To check the feasibility of the used scenarios as well as the participants’ usage of these visual stimuli (zebra crossing, bus station, playground), we additionally evaluated for each participant whether the mental model of the driving scene, the perceived attention demand, and the attention ratio were adapted from the baseline to each precursor scenario. A positive adaptation was assigned if the peripheries received a higher overall rating, rank, or attention ratio. Some participants did not change their behavior at all while others also showed negative adaptations by prioritizing the center over peripheral areas in the precursor scenarios compared to the baseline.

#### Software

Eye tracking data was processed using the software D-Lab [59]. Pupil recognition was postprocessed manually to achieve maximum data availability (Min = 87.08%) and attention ratio was calculated within the software. Data preparation and analysis was performed with RStudio [60] and Microsoft Excel [61].

## Results

### Mental model of vision

**Fig 6:**
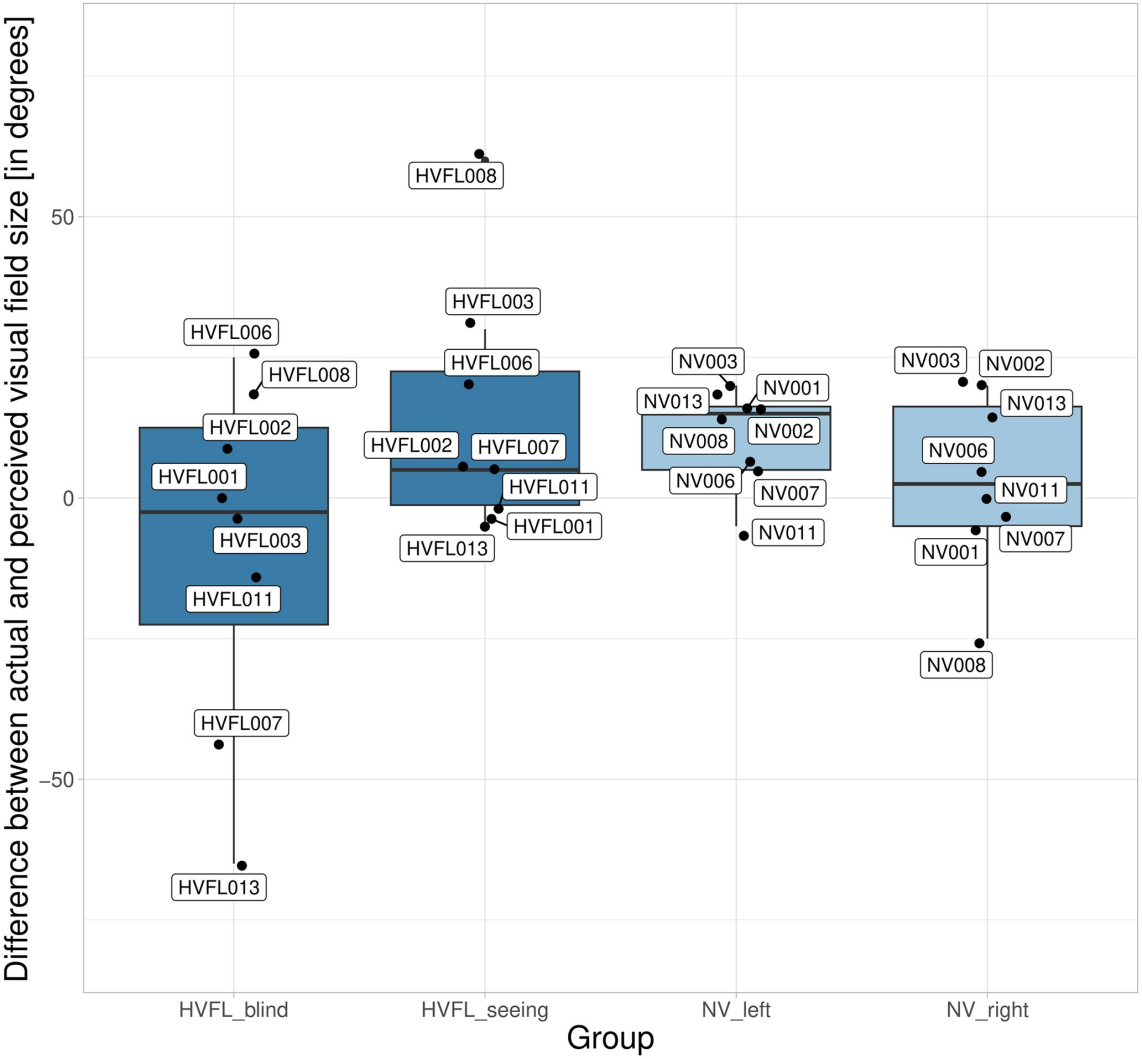
Difference between the actual and the perceived visual field size. Data is displayed per group (NV; HVFL) and side (left; right / blind; seeing) [in degrees]. Negative values indicate an overestimation of the visual field.

Concerning the difference between the subjective and objective visual field size (Fig 6), HVFL participants showed greater variance on their blind side (Mdn = -2.50°; IQR = 35°) compared to their seeing side (Mdn = 5.00°; IQR = 23.75°). NV participants showed less variance regarding their visual field on the left (Mdn = 15.00°; IQR = 11.25°) and on the right side (Mdn = 2.50°; IQR = 21.25°). These values must be interpreted considering extreme outliers in all conditions. The medians show an underestimation of the visual field in all conditions except for HVFL participants on their blind side, where the median value indicated a slight overestimation of 2.50°. As visible in Fig 6, participants HVFL007 and HVFL013 had an abnormal overestimation of their visual field on the blind side that largely differed from the rest of the sample. Potential explanations for their poor mental model of the visual field size include the finding that these two participants had the shortest time since onset to adjust their mental model (10 and 33 months), regularly used prism glasses, and failed to verbalize their visual impairment properly. Instead of explaining the missing visual input as all other participants did, they mentioned the resulting difficulties and frustrations. HVFL013 was also the only participant with no prior rehabilitation therapy and the oldest participant at 83 years.

**Fig 7:**
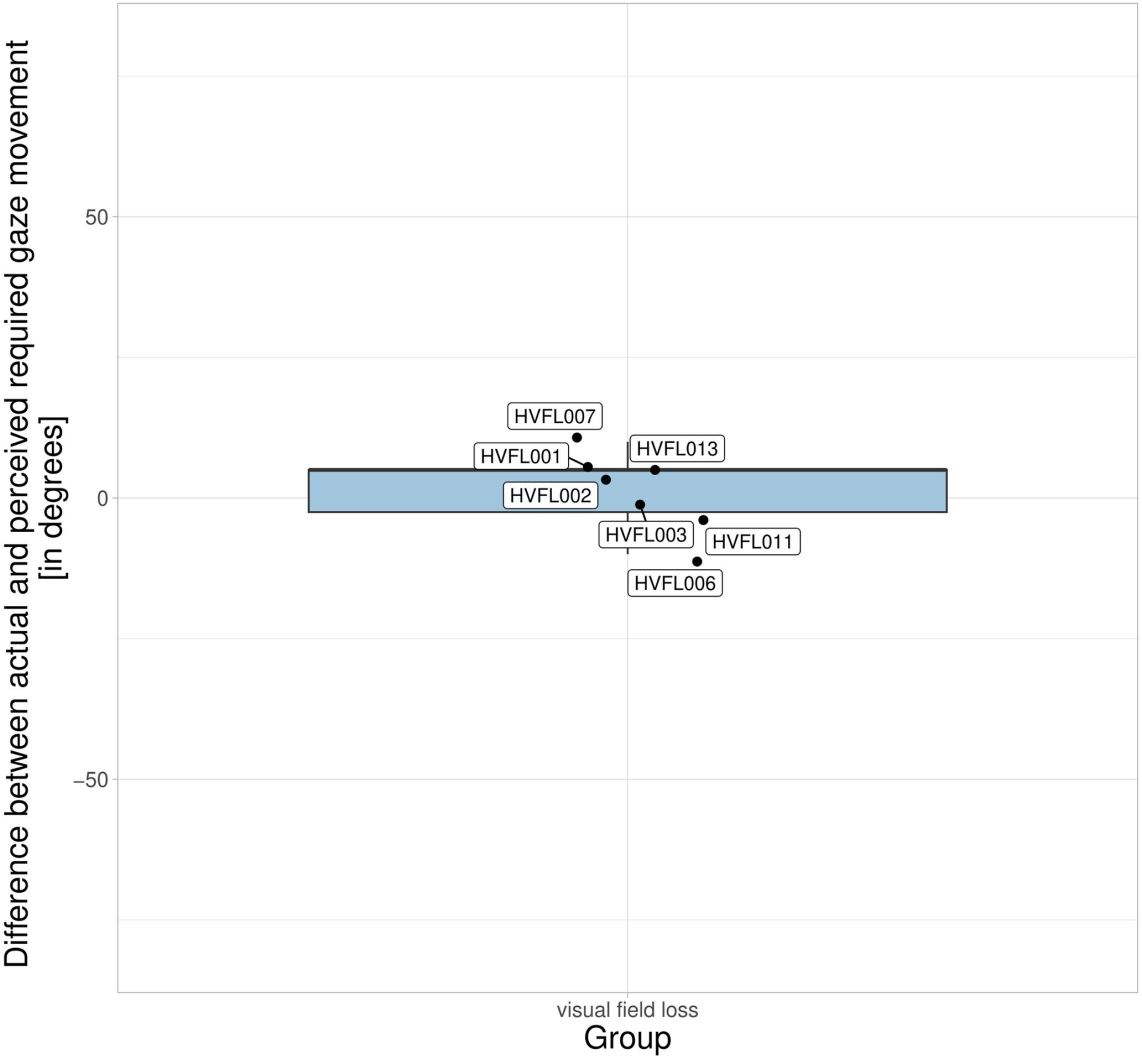
Difference between the actual and the perceived gaze movement required to perceive an object at an eccentricity of 30°. Negative values indicate an underestimation of the required gaze movement.

The mental model of the required gaze movement to perceive objects in the blind visual field showed little variation within HVFL participants (M = 1.43°; SD = 6.90°) with no extreme outliers and a slight median overestimation of 5.00°. Fig 7 shows that two participants HVFL006 and HVFL011 underestimated the required gaze movement but showed no large difference from the rest of the sample. Those two participants were inconspicuous in the mental model of the visual field size. Participants with an abnormal underestimation of the extent of the HVFL showed the greatest overestimation of the required gaze movement, contrasting the assumption that these two concepts build on each other (see Fig 1). The correlation between the two aspects of the mental model of vision was low (r(6) = 0.26), according to the thresholds proposed by Akoglu [62].

### Mental model of the driving scene

**Fig 8:**
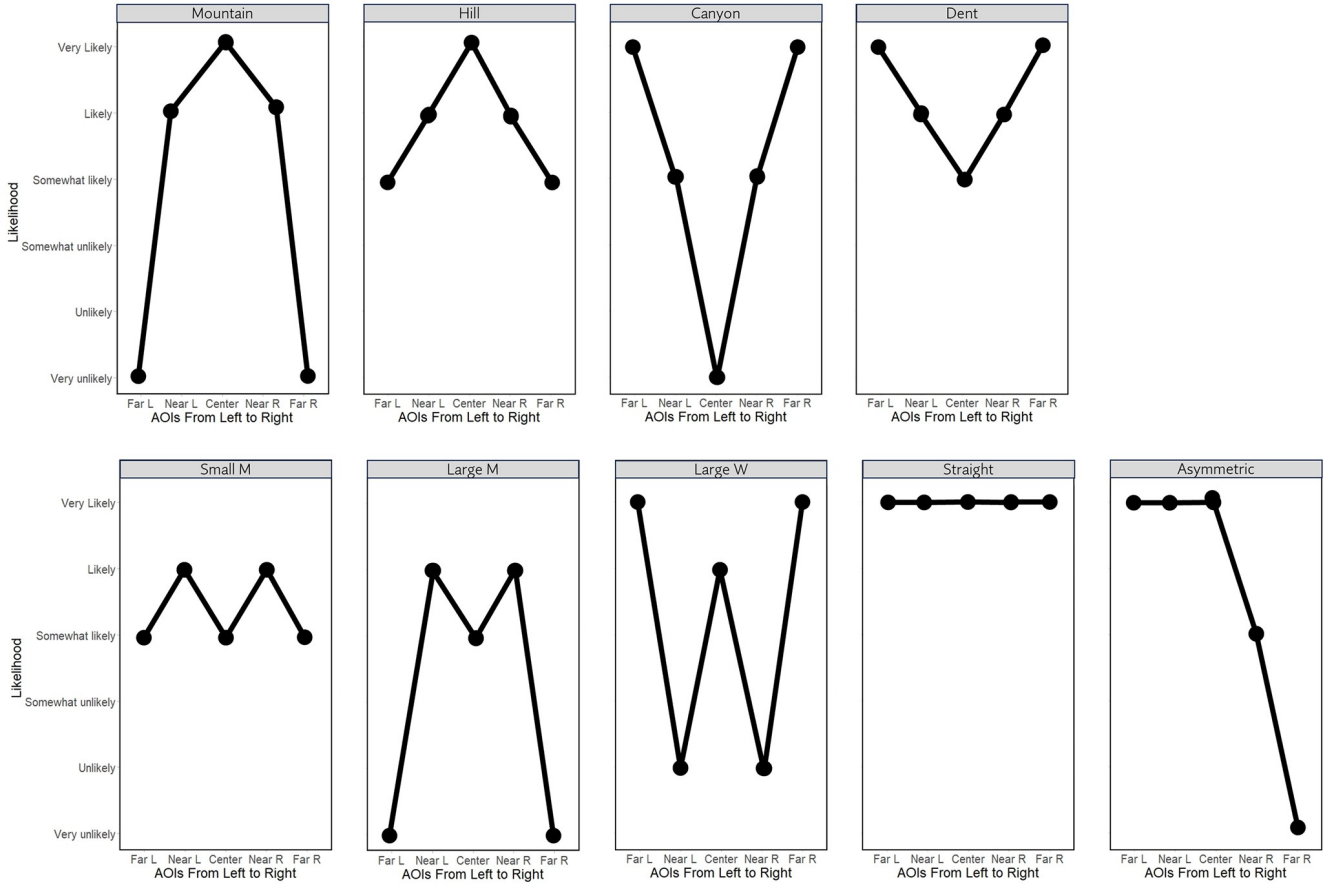
Depiction of the existent patterns in the mental model of the driving scene.

After watching the videos of the driving scenes, most of the participants stated they had experienced the scenarios, and only one participant HVFL002 did not remember any of the scenes. The precursors in the precursor scenarios were misunderstood or not noticed by three participants in the bus station scenario (two HVFL), one participant in the playground scenario (HVFL), and six participants in the zebra crossing scenario (five HVFL). The median scores for the AOI-overarching likelihood of hazards showed that participants rated all scenarios as “somewhat likely” but with two exceptions. HVFL participants had higher likelihood ratings for the zebra crossing (“likely”), and the same median rating was evident for NV participants concerning the playground scenario. On an individual level, we found nine different rating patterns for the five AOIs (see Fig 8). Two types gave a decreasing likelihood for more peripheral AOIs compared to the center (“Mountain” and “Hill” depending on whether the ratings ranged from positive (“(very/somewhat) likely”) to negative (“(very/somewhat) unlikely” or not) and two patterns increased the likelihood for peripheral areas (“Canyon” and “Dent”). Another pattern assigned the highest likelihood to the near periphery (“Small M”; no “Large M” in the data set), and two patterns gave the lowest rating to the near peripheral areas (“Large W” and “Small W”). Lastly, some participants gave equal ratings to all AOIs (“Straight”) or asymmetric likelihoods for the left and right sides (“Asymmetric”). As can be seen in Table 1, there was a tremendous interpersonal variation of patterns even in the most basic baseline scenario. HVFL participants showed greater stability in their mental models in different scenes compared to NV participants. Seven out of eight HVFL participants strongly favored the patterns “Hill” or “Mountain”. In the case of asymmetric patterns, one half still coincided with these patterns while the other side resembled “M”, “W” or “Straight” patterns. We found only five out of nine patterns among the HVFL participants (“Mountain”, “Hill”, “Large W”, “Canyon”, and “Asymmetric”). In comparison, NV participants showed eight of nine patterns and frequently multiple patterns within one person. The “Asymmetric” pattern was more frequent among HVFL than NV participants. HVFL003 was extraordinary because this participant was the only one with a consistent “Canyon” pattern in the HVFL group. For the HVFL group, between two and three participants showed positive adaptations from the baseline to the precursor scenarios. This was higher for the NV group, with ranges between three and seven participants showing good adaptations, with the maximum at the playground scenario.

**Table 1:** Distribution of patterns for the mental model of the driving scene per participant and scenario.

	Baseline	Zebra crossing	Bus station	Playground
*HVFL*001	Mountain	Mountain	Mountain	Mountain
*HVFL*002	Hill	Hill	Hill	Hill
*HVFL*003	Canyon	Canyon	Canyon	Canyon
*HVFL*006	Large W	Mountain	Asymmetric	Mountain
*HVFL*007	Asymmetric	Mountain	Asymmetric	Mountain
*HVFL*008	Hill	Hill	Asymmetric	Hill
*HVFL*011	Mountain	Asymmetric	Mountain	Mountain
*HVFL*013	Mountain	Hill	Mountain	Hill
				
*NV*001	Mountain	Hill	Mountain	Mountain
*NV*002	Small M	Large M	Large M	Large M
*NV*003	Hill	Mountain	Mountain	Dent
*NV*006	Asymmetric	Straight	Large M	Canyon
*NV*007	Mountain	Hill	Mountain	Hill
*NV*008	Canyon	Small M	Mountain	Canyon
*NV*011	Large M	Large M	Large M	Canyon
*NV*013	Hill	Hill	Hill	Hill

### Perceived attention demand

**Fig 9:**
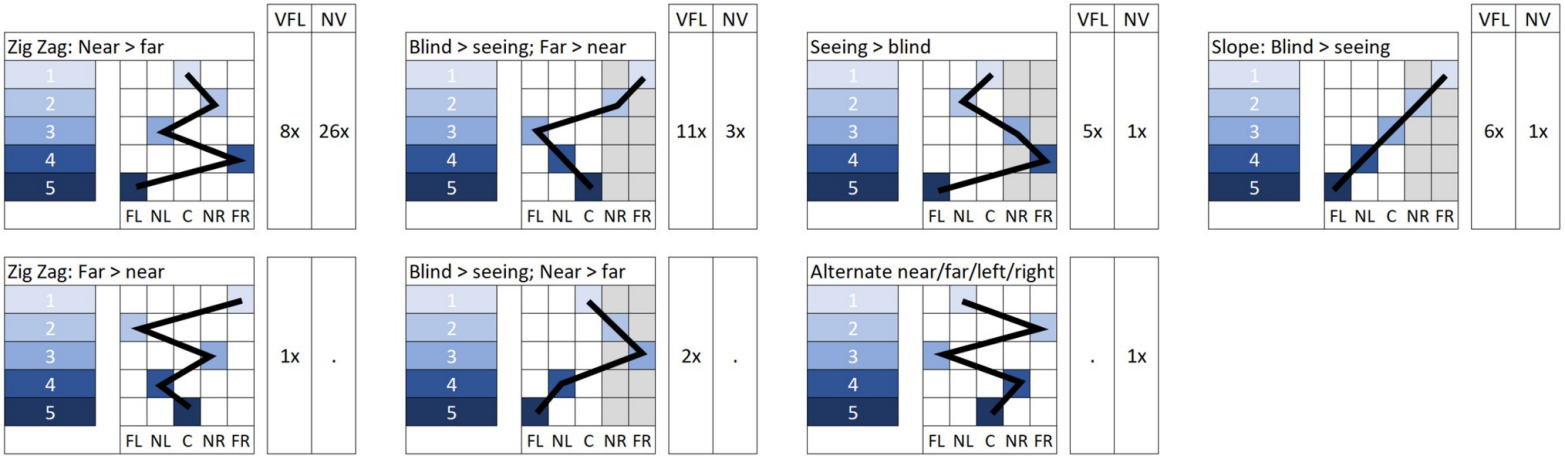
Display of patterns ranking the perceived attention demand. Each pattern is defined by the ranking of the five AOIs (far left (FL), near left (NL), center (C), near right (NR) and far right (FR)). The occurrence among both groups is indicated. For side-specific patterns, the blind side (in this figure right side as an example) is marked grey. Note that distinctions between the blind and seeing sides do not apply to NV participants.

Rankings for the perceived attention demand produced four frequently shown patterns and three additional patterns with only singular occurrences. Patterns and frequency of occurrence per group can be found in Fig 9. Participants with HVFL showed a great variety of patterns. NV participants mostly ranked the AOIs in a Zig Zag pattern with higher ranks for both near peripheries than far peripheries and varying rankings of the center. Four of eight HVFL participants and five of eight NV participants showed the same pattern over all scenarios. The number of participants with positive adaptations to the precursor scenarios varied between three and five for HVFL participants and between zero and five for NV participants. Most positive adaptations were found in the zebra crossing scenario for HVFL participants and the playground scenario for NV participants.

No link between individual patterns was evident concerning the scenario’s influence on the perceived attention demand. On a group level, the mean match was higher for NV than HVFL participants in all conditions (see Table 2). There were 23 cases with less than 50% rank matches among drivers with HVFL. In 16 of those, low matches stemmed from prioritizing the blind side in the perceived attention demand, but not the mental model of the driving scene. Only in four cases was the blind side not prioritized. The corresponding participants were HVFL007 and HVFL013, who overestimated the field of view on the blind side. It should be noted that for HVFL013, the influence of the poor mental model of the visual field size was highly scenario-specific in that they had high matches with good adaptation in the zebra crossing and playground scenario but low matches with a prioritization of the seeing side for the baseline and bus scenario. The two participants with an underestimation of the required gaze movements did not stand out in the perceived attention demand.

**Table 2:** Mean and standard deviation (in brackets) of the match of rank orders between the mental model of the driving scene and the perceived attention demand.

	Baseline	Zebra crossing	Bus station	Playground
*HVFL*	7.13 (2.17)	6.13 (3.09)	6.00 (3.12)	7.38 (3.07)
*NV*	9.75 (0.71)	9.38 (1.19)	9.00 (1.77)	8.63 (2.88)

The maximum possible match value was 10.

### Attention ratio

There was a closer relation between subjective measures (mental model of the driving scene and perceived attention demand, see Table 2) than between subjective and objective measures (perceived attention demand and attention ratio, see Table 3). The latter showed a higher match for the HVFL participants than for the NV participants. When looking at the direct impact of vision on the actual attention ratio, participants HVFL007 and HVFL008 failed to prioritize the blind over the seeing side in five scenarios. HVFL007 had a poor mental model of the visual field size and prioritized the seeing side in the perceived attention demand as well. HVFL008 had a comparatively smaller HVFL extent that was classified as quadrantanopia instead of hemianopia. However, participants with an underestimation of the required gaze movement and HVFL013, who was the only other participant with a poor mental model of the visual field size and quadrantanopia, behaved similarly to other participants. Positive adaptations of the attention ratio from the baseline to the precursor scenarios were found more frequently for NV participants (between six and eight participants) than the HVFL group (between three and five participants), with the highest number of good adaptations found in the playground and zebra crossing condition, respectively. It is particularly interesting for HVFL participants whether adaptations were made on the seeing, the blind, or both sides and to identify whether more attention on one side reduced attention on the opposite side. In the 24 scenarios experienced by eight participants in three precursor scenarios, a positive adaptation on the seeing side came with a reduced attention on the blind side three times. The reverse direction was never found. Negative adaptations in terms of a less attention on peripheral areas in the precursor compared to the baseline scenarios were only evident twice on the seeing side, but eleven times on the blind side.

**Table 3:** Mean and standard deviation (in brackets) of the match of rank orders between the perceived attention demand and the actual attention ratio.

	Baseline	Zebra crossing	Bus station	Playground
*HVFL*	6.79 (2.08)	6.25 (1.19)	7.25 (2.25)	5.88 (2.17)
*NV*	5.29 (2.44)	6.13 (3.09)	6.88 (1.96)	4.63 (2.39)

The maximum possible match value was 10.

### Driving performance

**Fig 10:**
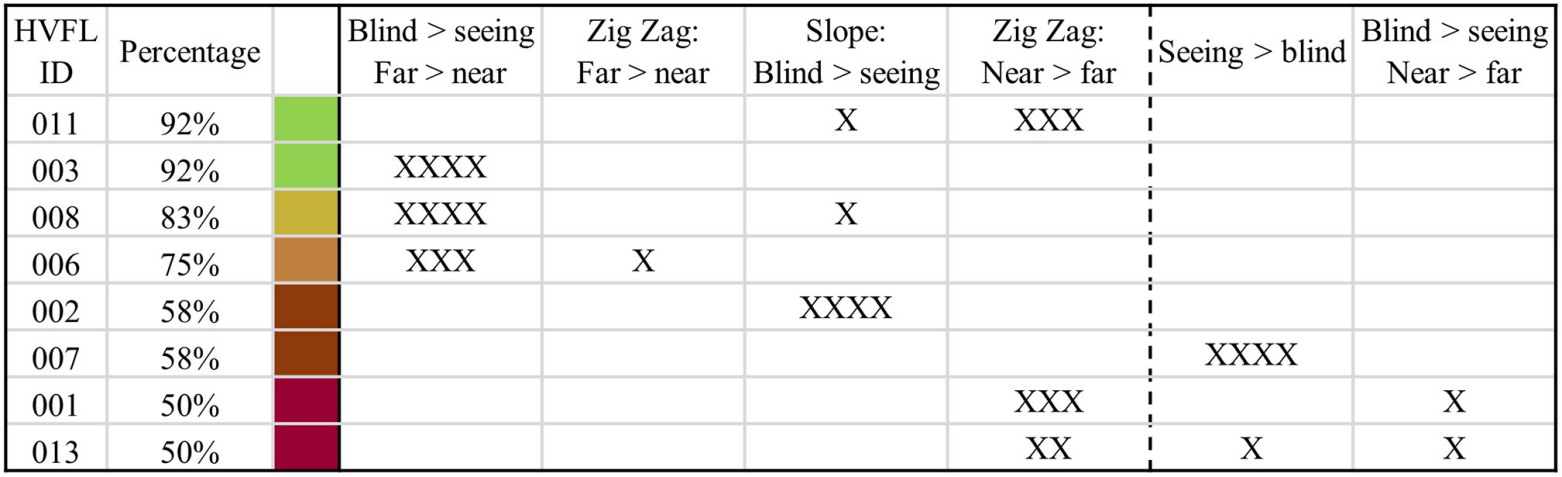
Ratio of collision free scenarios per HVFL participant (over all scenarios) and the relation to the underlying patterns in the test of the perceived attention demand. Each “x” represents one occurrence of the respective pattern in the baseline, zebra crossing, bus station, or playground scenario. Only scenarios with a crossing hazard are considered.

HVFL participants showed critical performance in terms of collisions or post-encroachment times below 1s in 30 of 48 scenarios (62.50%) when hazards appeared from their blind side and in 17 of 48 scenarios (35.42%) when hazards came from their seeing side. In 20 and 9 of those critical scenarios on the blind and seeing sides, respectively, a collision with the crossing cyclist occurred. No participant with HVFL was able to avoid collisions in all scenarios. Two participants only had one collision but had a critical post-encroachment time in three or four further scenarios. By comparison, only one collision occurred among NV drivers in the first drive and four further participants had one incident with a post-encroachment time under 1 second each. A closer look at the performance of HVFL participants in different conditions showed no overall difference between the order of the drives (15, 15, and 17 critical incidents in the first, second and third drive). The worst performance among HVFL was found in the playground scenario where 56.25% of scenarios (9 out of 16) had critical incidents compared to 37.50% in the bus station and zebra crossing each. As seen in Fig 10, some patterns from the perceived attention demand were correlated with the performance and most clearly the occurrence of crashes with the crossing cyclist. Specifically, patterns prioritizing the seeing side over the blind side or near peripheries over far peripheries were associated with more collisions.

## Discussion

### Mental model of vision

The mental model of the visual field size had a greater difference from the actual extent for participants with HVFL, especially on the blind side, as indicated by a large variance with great outliers. The gross overestimation of the visual field on the blind side that two participants with HVFL displayed can be considered a risk factor for inadequate scanning on the blind side. The mental model of vision impacted the perceived attention demand and the actual gaze distribution. Those participants with an abnormal overestimation of the field of view on the blind side tended to prioritize peripheral areas on the seeing side over the blind side. The mental model of the required gaze movements to perceive objects on the blind side yielded a small variance within the sample and did not affect the scanning. In the past, researchers have found that spatial representation is affected by impairments of the peripheral field of view, resulting in more placement errors and a compression of space [63–65]. This could explain the increased difficulties of participants with HVFL to judge the spatial extent of their peripheral impairment. The dissociation between perception and awareness in some participants with hemianopia due to anosognosia, blindsight, sightblindness, and visual hallucinations has also been elaborated by Chokron et al [66]. Koehler et al. [67] even point out that the awareness of one’s visual deficit can indicate the location and size of the underlying brain injury. In the presented study, participants with a severe overestimation of the field of view on the blind side had a short period of time since the onset of the HVFL, regular usage of prism glasses, and an insufficient understanding and verbalization of their HVFL. A short period of time since onset as found in this study equals less time to properly adjust the mental model to the new visual field extent and has been proposed as a factor relating to compensatory abilities in many studies [18]. The other potential causes should be investigated further as verbalizations could be a focus of targeted trainings and might be particularly indicated in prism users to avoid distortions in the mental models of the visual field. Surprisingly, we did not find any connections between the mental model of the required gaze movement and the other concepts in the theoretical model. This is most likely due to methodological issues, as participants had to point out the required gaze movement immediately after directly looking at the object of interest. It can be argued that this task does not actually draw on internal representations but relies largely on memory. In addition, we found that most of the participants did not manage to point toward their subjective midline straight-ahead and even had fluctuations between the left and right arms. An issue contributing to this deviation is the misalignment of the shoulder joint and entire arm from the horizontal and vertical position of the straight-ahead central gaze position that needs to be accommodated in the task. The results from the visual field size experiment nevertheless give a positive indication of this approach’s feasibility. However, future research should investigate such fluctuations under repeated task performance to evaluate its reliability. Applications of this method in the future should be aware that participants need to have sufficient mobility in their arms and no vertigo when standing with blindfolded eyes.

### Mental model of the driving scene

The mental model of the driving scene was more diverse among the NV participants than among the HVFL participants, both interindividually and intraindividually. All HVFL participants except one exhibited patterns with a continuous decrease in hazard likelihoods from central to peripheral AOIs. Although nine different patterns were found overall, judging their appropriateness or correctness is difficult. Since participants with contrasting patterns in the mental model of the driving scene (e.g., patterns “Mountain” and “Canyon” that allocate decreasing and increasing hazard likelihoods to peripheral areas, respectively) can lead to equal performances, it cannot be stated that one pattern surpasses the other. Across the scenarios, NV participants better adapted their mental models to situations with precursors with increasing hazard likelihoods, especially in the periphery. Therefore, it seems that HVFL participants largely have a situation-overarching mental model of the driving scene that is adapted only in minor aspects when visible markers indicate an increased danger of crossing hazards. It could be argued that drivers with HVFL show less anticipatory behavior and use visual precursors less when forming their mental model of the upcoming scene. The little current driving experience and the more frequent lack of proper understanding of the scenarios compared to NV drivers support this notion. Furthermore, it would be in line with the theory that situation awareness is based on peripheral information [68, 69]. Another explanation for the low adaptation to the precursors could be that the ratings in the baseline condition already represented the maximum hazard likelihood HVFL participants would potentially attribute to the AOIs. Although individual AOIs received one of the highest ranks among many participants in the baseline as well as precursor scenarios, both groups only had a median rating of “somewhat likely” which would indicate some room for potential increases. Only the zebra crossing and the playground received higher median ratings from the HVFL and NV groups, respectively. It could be argued that the zebra crossing situation is one of the most common scenarios in road traffic to explain why participants with HVFL with little current driving experience are more sensitive to this scenario. On the other hand, bus stations with halting buses are also relatively common but were not rated exceptionally high by any group. The zebra crossing is the only scenario whose main characteristics are presented centrally, so participants with HVFL might react more strongly to that. From a methodological standpoint, the HVFL participants most often did not understand the zebra crossing correctly. When asked to describe the scenario, some did not mention the road markings or signs but instead assumed them to be indicators of an intersection. Increased peripheral hazard risks might stem from such a misinterpretation. The playground, on the contrary, is the only scenario that incorporates environmental and behavioral hazard predictions according to the definition by Crundall et al. [42]. The authors found that a greater distance between predictor and hazard can differentiate between novice and expert drivers since only the latter successfully makes the required association. Our results would contradict this finding if normal-sighted drivers are regarded as experts compared to HVFL drivers. However, three participants with NV remarked that this scenario seemed rather unusual. Therefore, the scenario’s design might have been exaggerated or unrealistic, so it alerted the participants with more driving experience. Regarding the proposed connection between the mental model of the driving scene and other constructs, we found similar sensitivities toward the zebra crossing and the playground scenario in the subjectively perceived attention demand and the actual attention ratio among HVFL and NV participants, respectively. On an individual level, we did not find differences in the perceived attention demand that can be directly linked to the underlying mental model of the driving scene. However, the overall match between the mental model of the driving scene and the perceived attention demand was high, especially for NV participants. This finding aligns with the theory by Biebl et al. [17] that the perceived attention demand is based on a mixture of the mental model of vision and the mental model of the driving scene. Since no one-sided vision impairments must be incorporated in NV drivers, the perceived attention demand should be almost identical to the mental model of the driving scene, which is what we found. Lower match rates among HVFL participants were mostly due to the incorporation of the HVFL in the perceived attention demand, but not the mental model of the driving scene. However, it should be noted that methodological necessities might also contribute to very high matches of the NV participants. In the mental model of the driving scene, NV participants frequently showed patterns where multiple AOIs received an identical rank so that only two or three rank levels resulted. This inherently allows for more diverse patterns in the perceived attention demand ranking to match the ranks in the mental model of the driving scene, producing high match values. On a broader note, evaluating the mental model of the driving scene and the perceived attention demand was a novel measurement approach. We found that the participants required sufficient cognitive abilities to understand the rather abstract type of questioning and, especially, the differentiation between the two measures. Individual participants who presented with cognitive impairments and were later excluded from the analysis were unable to understand the instructions accordingly. We found that most participants valued the hazard likelihood of the peripheral areas symmetrically while incorporating the visual field loss only in the perceived attention demand as intended. However, the “asymmetric” pattern in the mental model of the driving scene only presented itself once among the NV participants and five times in the HVFL group. Future applications should be aware of the large individualization of patterns, which may necessitate analyses on an individual level.

### Actual attention ratio

Generally, the subjectively perceived attention demand was less closely related to objective gaze data than to the subjective measure of the assumed hazard likelihoods. On a group level, however, HVFL participants seemed to base their attention allocation more on the top-down generated theoretical attention demand than NV participants did. NV participants frequently ranked the far peripheries low in both subjective measures but scanned them appropriately in the actual drives and especially the scenarios with precursors. Some patterns expressed by the NV and HVFL participants also ranked the center very low in both subjective measures. This represents the considerable individuality of answering the questions where some found the center to be most hazardous and others the peripheries. Since different valid deliberations can lead to these conclusions, neither should be considered to be better or worse. However, in the actual drive, allocating much attention to the center is inevitable for vehicle stabilization, resulting in a low match with the subjective measures. Next to these methodological considerations, the closer dependence of gaze behavior on the top-down processes evaluated in the subjective measures could substantiate the theory proposed by Biebl et al. [17] and evaluated in this paper. An increase in attention toward peripheral areas in the scenarios with visual precursors compared to the baseline was found more frequently for NV than for HVFL participants with the group-specific sensitivity to the playground and zebra crossing scenario, respectively. Therefore, it seems that NV participants can better use visual markers to anticipate hazards and adapt their scanning behavior. Interestingly, participants with HVFL showed more bad adaptations on the blind side than the seeing side in terms of lower attention in the precursor scenarios. This might indicate that participants with HVFL did not disregard the precursors but instead did not manage appropriate adaptations potentially due to the increased workload of processing the visual precursors.

### Driving performance

The large number of critical incidents among the HVFL group on both sides supports the idea that many HVFL participants experienced difficulties managing the scenarios. The time to collision of the approaching cyclist of 5 seconds should have been sufficient to avoid collisions as it has been proposed as the threshold for warning systems [70, 71], equals double the perception-response time of 2.5 seconds [72] and has been used by other researchers with similar scenarios in the past [26]. The appropriateness of the timing is further supported by the good performance of NV participants, who avoided a collision in all but one scenario and only had four further critical incidents. The result that no participant with HVFL safely maneuvered all scenarios is surprising considering reports [5, 8, 21, 22] that between 14% and 77% show high driving performances due to successful compensation. The considerable surplus of critical occurrences on the blind compared to the seeing side is however in line with those reports. One factor contributing to the bad performance in this study could have been the little driving experience, where only two participants with HVFL drove regularly. However, since those participants were not the best performers, the influence of their driving experience is questionable. We found that neither the mental model of the driving scene nor the mental model of vision alone could predict performance. However, participants with an overestimated visual field on the blind side were in the lower half of performers. On the other hand, the combination of mental models in the perceived attention demand correlated with the resulting performance, where patterns prioritizing the seeing side or near peripheries over far peripheries were related to collision more frequently.

### Limitations

Overall, the presented study came with several limitations. The final sample consisted of sixteen participants, eight with and eight without HVFL, because numerous participants had to be excluded to avoid influences from cognitive impairments, neglect, or lack of driving experience. Although it is advised to have a controlled sample rather than a large sample in research where comorbidities can heavily influence the results, interpretations should be drawn with caution. However, the small sample allowed for an in-depth analysis of the data at a highly individual level, which is warranted when investigating the highly individual top-down mechanisms leading to compensatory scanning. As a pilot study, we recommend replication with a larger sample. The exploratory and descriptive approach is recommended to understand the mechanisms of compensation. Much research has previously shown significant interindividual differences in scanning and compensatory mechanisms, and the classical approach of inferential statistics has not yielded satisfactory clarification of the underlying processes [6, 8–12, 25, 26, 73, 74]. As mentioned, the HVFL participants’ sample mainly consisted of current non-drivers. However, this might be of specific interest since any rehabilitation program, training, or assistance system should tackle the status quo. In many jurisdictions, persons with homonymous hemianopia are currently excluded from driving [2, 3]. In addition, investigating persons who have not driven in years is a worst-case approach as the modulation of top-down processes and behavioral adaptations is not only influenced by time but more heavily by experiences [29]. Lastly, the scenario design should be improved using real-world observations of crossing hazards to refine the proximity to the simulation’s reality and optimize the hazards’ timing.

## Conclusion

Three novel measures for the top-down processes of drivers with HVFL were developed and tested. The spatial measurement of the mental model of the visual field size served as a useful tool to identify individual participants who had a severe overestimation of their visual field (as indicated by cluster analysis). This resulted in the prioritization of the seeing over the blind side in other subjective and objective measures. Measuring the dissociation between perception and awareness could be a valid tool to predict driving safety, as indicated by the results of this pilot study. On the contrary, the measure for the mental model of required gaze movements should be revisited as it was most likely based on memory instead of the targeted spatial representations. The measures for the mental model of the driving scene and the perceived attention demand produced highly individual findings, which complicates data analysis and demand considerations on an individual level. Due to the high number of individual patterns, a sufficient sample size is advised to receive robust results. While absolute evaluations of the patterns are hardly possible, these measures can be useful to compare groups or scenarios and should be rated considering the resulting driving performance. The pilot study indicated that subjectively prioritizing attention on the seeing side over the blind side and near peripheries over far peripheries leads to more collisions. We found that HVFL participants used visual precursors less for hazard anticipation, both subjectively and objectively. The data furthermore yielded promising indicators for the theoretical model of requirements for compensatory scanning as postulated by Biebl et al. [17]. The perceived attention demand closely matched the mental model of the driving scene and only deviated from it to consider the blind visual field. The translation of the subjective attention demand to the actual gaze distribution was not as direct due to methodological issues but still showed the same group-wise sensitivities towards individual scenarios. We found indications that HVFL participants relied more heavily on their top-down assessments when scanning than NV participants, as proposed by Biebl et al. [17]. This pilot study yielded promising results on the newly developed methods to elaborate the top-down mechanisms in drivers with HVFL. Since prior approaches using inferential statistics have not fully accounted for the individuality and mechanisms behind compensation, these measures could be a valuable tool to elucidate the black box behind the behavior. This is important to achieve inclusive mobility as trainings targeting poor awareness of the visual field size or unsuitable prioritizations of different areas in a driving scene could improve the driving safety of current non-drivers with HVFL. Driver assistant systems could also provide more targeted and individualized support if the mechanisms behind insufficient scanning are understood better. Future studies should focus on further optimizing and evaluating these methods regarding reliability and validity and revisit the verification of the postulated requirements for successful compensatory scanning with a larger sample.

## Supporting information

S1 Fig HVFL characteristics. (PDF)

S2 Fig Difference between the perceived and actual extent of the visual field and the required gaze movement to perceive objects in the blind visual field. Measurement for the mental model of vision.
(PDF)

S3 Fig Recognition and understanding of scenarios. (PDF)

S4 Fig Subjective likelihood of hazard information being visible in the AOIs. Five AOIs were evaluated (far left (FL), near left (NL), center (C), near right (NR), far right (FR)) per scenario. Likelihood was indicated on a six-point Likert scale (“very unlikely” (1) to “very likely” (6)). Measurement for the mental model of the scene.
(PDF)

S5 Fig Ranking of the perceived attention demand in the AOIs. Five AOIs were evaluated (far left (FL), near left (NL), center (C), near right (NR), far right (FR)) per scenario.
(PDF)

S6 Fig Attention ratio for the AOIs. Five AOIs were evaluated (far left (FL), near left (NL), center (C), near right (NR), far right (FR)) per scenario.
(PDF)

S7 Fig Post-encroachment time per scenario and side of the crossing cyclist. (PDF)
