# Supplementary material for: Knowing me, knowing you—A study on top-down requirements for compensatory scanning in drivers with homonymous visual field loss
Source: PLoS One. 2024 Mar 1;19(3):e0299129. doi: 10.1371/journal.pone.0299129 (PMC10906860; doi:10.1371/journal.pone.0299129)
Supplement: S1 Fig — (PDF) [file pone.0299129.s001.pdf]

Group	Time since onset of HVFL [in months]	Side of HVFL	Type of HVFL
HVFL001	241.00	Right	Hemianopia
HVFL002	34.00	Right	Hemianopia
HVFL003	47.00	Left	Hemianopia
HVFL006	59.00	Right	Hemianopia
HVFL007	33.00	Left	Hemianopia
HVFL008	52.00	Left	Quadrantanopia
HVFL011	215.00	Left	Hemianopia
HVFL013	10.00	Right	Quadrantanopia
