# Supplementary material for: Knowing me, knowing you—A study on top-down requirements for compensatory scanning in drivers with homonymous visual field loss
Source: PLoS One. 2024 Mar 1;19(3):e0299129. doi: 10.1371/journal.pone.0299129 (PMC10906860; doi:10.1371/journal.pone.0299129)
Supplement: S2 Fig — Measurement for the mental model of vision. (PDF) [file pone.0299129.s002.pdf]

ID	Begin HVFL	End seeing side	Gaze movement
HVFL001	0.00	-5.00	5.00
HVFL002	10.00	5.00	5.00
HVFL003	-5.00	30.00	0.00
HVFL006	25.00	20.00	-10.00
HVFL007	-45.00	5.00	10.00
HVFL008	20.00	60.00	NA
HVFL011	-15.00	0.00	-5.00
HVFL013	-65.00	-5.00	5.00

ID	End left side	End right side
NV001	15.00	20.00
NV002	5.00	5.00
NV003	15.00	-5.00
NV006	5.00	-5.00
NV007	15.00	-25.00
NV008	20.00	20.00
NV011	20.00	15.00
NV013	-5.00	0.00
