# Supplementary material for: Knowing me, knowing you—A study on top-down requirements for compensatory scanning in drivers with homonymous visual field loss
Source: PLoS One. 2024 Mar 1;19(3):e0299129. doi: 10.1371/journal.pone.0299129 (PMC10906860; doi:10.1371/journal.pone.0299129)
Supplement: S3 Fig — (PDF) [file pone.0299129.s003.pdf]

ID	Experienced scenario				Understood scenario		
	Baseline	Zebra crossing	Playground	Bus	Zebra crossing	Playground	Bus
HVFL001	no	yes	yes	yes	no	no	yes
HVFL002	no	no	no	no	no	yes	yes
HVFL003	no	yes	yes	yes	yes	yes	yes
HVFL006	yes	yes	yes	yes	yes	yes	no
HVFL007	yes	yes	yes	yes	no	yes	yes
HVFL008	yes	yes	yes	yes	no	yes	yes
HVFL011	yes	yes	yes	yes	yes	yes	yes
HVFL013	yes	yes	yes	yes	no	yes	no
NV001	yes	yes	yes	yes	yes	yes	yes
NV002	yes	yes	yes	yes	yes	yes	yes
NV003	yes	yes	yes	yes	yes	yes	yes
NV006	yes	yes	yes	yes	yes	yes	yes
NV007	yes	yes	yes	yes	yes	yes	yes
NV008	yes	yes	yes	yes	yes	yes	yes
NV011	yes	yes	yes	yes	yes	yes	yes
NV013	yes	yes	yes	yes	no	yes	no
