# Supplementary material for: Knowing me, knowing you—A study on top-down requirements for compensatory scanning in drivers with homonymous visual field loss
Source: PLoS One. 2024 Mar 1;19(3):e0299129. doi: 10.1371/journal.pone.0299129 (PMC10906860; doi:10.1371/journal.pone.0299129)
Supplement: S4 Fig — Five AOIs were evaluated (far left (FL), near left (NL), center (C), near right (NR), far right (FR)) per scenario. Likelihood was indicated on a six-point Likert scale (“very unlikely” (1) to “very likely” (6)). Measurement for the mental model of the scene. (PDF) [file pone.0299129.s004.pdf]

ID	Baseline					Zebra crossing					Playground					Bus				
	FL	NL	C	NR	FR	FL	NL	C	NR	FR	FL	NL	C	NR	FR	FL	NL	C	NR	FR
HVFL001	2	3	6	3	2	2	5	6	5	2	2	4	6	4	2	2	4	6	4	2
HVFL002	4	5	6	5	4	4	5	6	5	4	4	4	6	5	5	4	4	6	5	6
HVFL003	6	5	2	4	4	6	4	2	4	6	6	4	2	4	6	6	4	1	4	6
HVFL006	6	3	6	3	6	3	5	6	3	1	3	4	6	4	2	5	4	6	4	1
HVFL007	2	5	5	3	4	1	5	6	5	1	1	4	5	4	2	1	5	6	2	4
HVFL008	5	5	6	5	5	4	5	6	5	5	5	5	6	5	5	4	6	6	6	6
HVFL011	1	4	5	4	1	2	5	4	4	2	1	4	6	4	1	1	3	6	4	4
HVFL013	3	3	6	5	4	NA	5	6	5	4	4	5	6	6	4	2	5	6	4	2
NV001	2	4	5	2	2	4	4	6	4	4	3	5	5	5	3	1	4	6	4	1
NV002	1	3	2	3	1	3	6	2	6	3	2	4	2	4	2	2	4	2	4	3
NV003	4	4	6	4	4	3	6	6	6	3	5	4	4	4	5	2	3	6	6	3
NV006	1	4	1	1	1	1	1	1	1	1	5	3	1	3	5	2	5	2	5	2
NV007	2	6	6	6	2	4	6	6	6	4	5	6	6	6	5	2	4	6	4	2
NV008	4	4	1	3	3	1	3	1	3	1	6	4	2	4	6	2	4	5	5	3
NV011	5	6	3	6	5	5	6	3	6	5	6	5	3	5	6	3	6	2	6	3
NV013	4	5	6	5	4	4	4	6	4	4	5	6	6	6	5	5	5	6	5	5
