# Supplementary material for: Knowing me, knowing you—A study on top-down requirements for compensatory scanning in drivers with homonymous visual field loss
Source: PLoS One. 2024 Mar 1;19(3):e0299129. doi: 10.1371/journal.pone.0299129 (PMC10906860; doi:10.1371/journal.pone.0299129)
Supplement: S5 Fig — Five AOIs were evaluated (far left (FL), near left (NL), center (C), near right (NR), far right (FR)) per scenario. (PDF) [file pone.0299129.s005.pdf]

ID	Baseline					Zebra crossing					Playground					Bus				
	FL	NL	C	NR	FR	FL	NL	C	NR	FR	FL	NL	C	NR	FR	FL	NL	C	NR	FR
HVFL001	5	3	1	2	4	5	4	1	2	3	5	3	1	2	4	5	3	1	2	4
HVFL002	5	4	3	2	1	5	4	3	2	1	5	4	3	2	1	5	4	3	2	1
HVFL003	1	2	3	5	4	1	2	5	4	3	1	2	5	4	3	1	2	3	5	4
HVFL006	3	4	5	2	1	3	4	5	2	1	2	4	5	3	1	3	4	5	2	1
HVFL007	5	4	1	3	2	5	3	1	2	4	5	3	1	2	4	5	3	1	4	2
HVFL008	1	2	5	4	3	1	2	3	5	4	1	2	5	4	3	1	2	3	5	4
HVFL011	4	2	1	3	5	4	1	2	3	5	4	2	1	3	5	1	2	3	4	5
HVFL013	5	2	1	3	4	5	3	1	2	4	5	2	1	3	4	5	4	1	2	3
NV001	5	2	1	3	4	5	2	3	1	4	5	2	3	1	4	5	3	1	2	4
NV002	5	3	2	1	4	5	2	3	1	4	5	2	3	1	4	5	3	1	2	4
NV003	4	3	1	2	5	5	3	1	2	4	1	5	2	4	3	4	3	1	2	5
NV006	3	1	5	4	2	4	2	5	1	3	2	4	5	3	1	4	2	5	1	3
NV007	5	3	1	2	4	5	3	1	2	4	5	3	1	2	4	5	3	1	2	4
NV008	1	2	5	3	4	5	1	3	2	4	2	4	5	3	1	5	4	1	2	3
NV011	3	1	5	2	4	3	1	5	2	4	1	3	5	4	2	3	1	5	2	4
NV013	4	2	1	3	5	4	2	1	3	5	4	2	1	3	5	4	3	1	2	5
