# Supplementary material for: Knowing me, knowing you—A study on top-down requirements for compensatory scanning in drivers with homonymous visual field loss
Source: PLoS One. 2024 Mar 1;19(3):e0299129. doi: 10.1371/journal.pone.0299129 (PMC10906860; doi:10.1371/journal.pone.0299129)
Supplement: S6 Fig — Five AOIs were evaluated (far left (FL), near left (NL), center (C), near right (NR), far right (FR)) per scenario. (PDF) [file pone.0299129.s006.pdf]

ID	Baseline (Drive A)					Baseline (Drive B)					Baseline (Drive C)					Zebra crossing					Playground					Bus				
	FL	NL	C	NR	FR	FL	NL	C	NR	FR	FL	NL	C	NR	FR	FL	NL	C	NR	FR	FL	NL	C	NR	FR	FL	NL	C	NR	FR
HVFL001	0.00	2.62	53.63	31.05	0.00	12.66	0.00	23.80	54.30	4.07	3.07	4.66	85.09	10.92	0.00	3.87	0.00	85.29	0.00	0.00	26.66	0.00	10.89	26.62	4.18	5.82	0.00	37.82	59.50	5.28
HVFL002	0.00	0.00	44.53	0.00	0.00	0.00	0.00	53.05	14.48	0.00	0.00	0.00	75.22	5.60	0.00	0.00	0.00	34.11	26.68	6.23	0.00	0.00	53.86	6.80	0.00	0.00	0.00	92.24	8.05	0.00
HVFL003	28.96	18.32	37.24	0.00	12.67	19.04	14.00	23.48	11.94	7.94	32.16	22.63	27.76	9.41	15.82	23.38	11.96	18.50	0.00	4.69	23.87	40.41	15.09	8.27	14.39	3.49	39.54	45.86	3.58	0.00
HVFL006	0.00	0.00	17.08	32.74	15.45	0.00	0.00	0.00	41.36	40.23	8.43	0.00	17.16	27.90	25.70	14.47	5.01	0.00	22.04	37.73	14.69	2.82	9.55	31.65	17.43	8.82	5.87	4.05	40.56	22.18
HVFL007	0.00	31.38	65.67	5.73	0.00	0.00	34.54	75.71	5.34	8.15	30.15	15.89	55.79	0.00	0.00	41.22	15.72	42.42	0.00	0.00	0.00	0.00	79.17	5.81	15.41	0.00	0.00	90.58	9.42	0.00
HVFL008	0.00	0.00	69.64	28.26	0.00	0.00	0.00	78.13	9.89	0.00	0.00	13.14	45.22	0.00	0.00	0.00	9.02	41.66	0.00	0.00	0.00	12.54	48.61	0.00	35.50	0.00	0.00	54.99	29.94	0.00
HVFL011	79.70	19.97	0.00	0.00	0.00	82.72	7.76	0.00	0.00	0.00	56.82	19.57	20.43	0.00	0.00	37.57	34.71	16.70	0.00	0.00	64.23	15.78	7.44	0.00	3.26	65.21	20.48	8.99	0.00	0.00
HVFL013	0.00	0.00	55.45	41.24	0.00	0.00	0.00	55.17	29.65	2.76	0.00	0.00	66.74	29.64	0.00	0.00	0.00	33.74	28.97	27.89	0.00	0.00	76.77	15.13	0.00	0.00	0.00	66.14	0.00	0.00
NV001	5.17	0.00	51.80	20.81	8.46	15.86	0.00	43.46	2.64	9.52	12.92	0.00	39.50	9.14	9.41	11.57	0.00	41.76	32.34	0.00	31.14	3.59	18.45	14.15	30.19	14.18	17.55	21.85	36.48	0.00
NV002	3.92	0.00	27.52	33.88	33.73	13.26	0.00	73.81	2.39	7.44	8.19	0.00	56.66	2.91	30.32	15.80	0.00	32.84	13.69	23.53	33.34	2.24	10.25	6.76	45.48	12.92	4.48	21.01	7.22	43.64
NV003	0.00	0.00	75.64	10.29	0.00	0.00	0.00	99.99	3.05	0.00	0.00	0.00	96.48	4.23	0.00	0.00	0.00	81.63	14.48	0.00	26.68	3.22	27.59	9.36	31.66	0.00	6.34	87.58	0.00	0.00
NV006	4.65	0.00	30.98	38.48	14.77	26.87	8.62	15.05	15.94	26.58	10.59	17.28	19.95	0.00	25.95	43.85	0.00	11.22	24.57	5.07	43.98	5.06	20.49	11.11	19.40	6.76	9.00	34.00	10.23	28.41
NV007	3.43	7.38	53.98	14.16	17.74	5.61	0.00	49.34	5.01	3.84	3.27	10.56	44.22	20.90	17.24	5.92	13.40	53.63	11.34	9.87	17.75	4.97	33.52	3.11	29.00	6.68	6.19	49.90	6.34	13.61
NV008	20.32	0.00	20.78	11.72	12.82	34.35	13.27	28.10	7.62	10.53	11.49	0.00	48.03	0.00	35.61	33.05	0.00	35.50	4.61	11.44	11.72	0.00	16.30	6.63	66.32	0.00	34.55	34.43	4.93	24.30
NV011	7.18	2.15	44.71	12.90	18.73	35.06	0.00	35.94	0.00	4.01	27.31	17.54	24.35	13.18	7.31	19.04	7.50	46.48	4.54	10.84	27.41	1.95	25.21	26.04	15.34	2.52	16.15	30.94	4.29	37.36
NV013	22.46	4.36	38.75	3.11	13.32	14.77	10.81	33.82	16.45	10.04	0.00	19.93	15.50	39.44	21.96	6.15	17.99	64.08	0.00	0.00	34.66	0.00	16.27	9.20	36.57	10.88	12.86	36.08	5.33	19.55
