# Supplementary material for: Knowing me, knowing you—A study on top-down requirements for compensatory scanning in drivers with homonymous visual field loss
Source: PLoS One. 2024 Mar 1;19(3):e0299129. doi: 10.1371/journal.pone.0299129 (PMC10906860; doi:10.1371/journal.pone.0299129)
Supplement: S7 Fig — (PDF) [file pone.0299129.s007.pdf]

ID	Post-encroachment time: Left side hazard					
	Baseline (Drive A)	Baseline (Drive B)	Baseline (Drive C)	Zebra crossing	Playground	Bus
HVFL001	Collision	0.60	0.78	1.82	1.20	1.70
HVFL002	0.66	3.06	4.58	3.62	3.20	Collision
HVFL003	1.02	1.48	2.02	1.98	Collision	1.30
HVFL006	3.32	3.78	2.42	3.44	2.42	2.68
HVFL007	0.06	Collision	4.30	3.58	Collision	0.34
HVFL008	Collision	Collision	Collision	1.54	1.16	1.44
HVFL011	Collision	Collision	Collision	Collision	0.48	0.34
HVFL013	0.12	0.04	2.32	3.26	5.08	3.56
NV001	Collision	2.72	2.08	2.88	2.08	2.50
NV002	2.28	1.96	2.18	2.50	2.88	2.02
NV003	2.20	2.40	1.42	2.12	0.92	2.24
NV006	3.00	3.80	4.32	3.90	3.08	2.74
NV007	2.08	1.70	2.74	3.06	2.40	2.60
NV008	1.46	1.10	3.22	2.08	1.14	1.52
NV011	1.52	1.10	2.32	1.90	1.38	2.40
NV013	2.12	1.18	1.90	4.30	3.06	2.56

ID	Post-encroachment time: Right side hazard					
	Baseline (Drive A)	Baseline (Drive B)	Baseline (Drive C)	Zebra crossing	Playground	Bus
HVFL001	Collision	Collision	0.84	Collision	Collision	0.60
HVFL002	4.66	Collision	4.44	3.46	Collision	Collision
HVFL003	1.62	1.36	0.96	1.60	1.06	1.39
HVFL006	Collision	Collision	1.82	3.40	Collision	1.50
HVFL007	3.32	4.80	3.50	7.04	Collision	3.48
HVFL008	0.26	2.36	Collision	1.80	0.18	1.10
HVFL011	0.34	0.90	1.34	1.58	Collision	1.84
HVFL013	Collision	0.26	0.80	Collision	Collision	Collision
NV001	2.78	1.90	2.88	3.76	3.30	1.78
NV002	2.68	0.58	1.92	2.40	2.78	2.02
NV003	2.42	2.58	1.64	1.88	1.18	1.32
NV006	3.42	2.88	3.86	5.18	3.80	4.02
NV007	3.08	3.26	1.62	2.68	1.64	2.22
NV008	1.94	1.92	2.02	1.62	2.42	1.88
NV011	0.64	1.50	1.22	1.74	1.88	2.16
NV013	3.00	0.42	1.94	2.72	2.10	3.60
